# Neonatal SARS-CoV-2 infection

**DOI:** 10.6061/clinics/2020/e1996

**Published:** 2020-05-25

**Authors:** Werther Brunow de Carvalho, Maria Augusta Cicaroni Gibelli, Vera Lucia Jornada Krebs, Valdenise Martins Laurindo Tuma Calil, Carla Marques Nicolau, Cíntia Johnston

**Affiliations:** I Terapia Intensiva em Neonatologia/Pediatria, Departamento de Pediatria, Faculdade de Medicina FMUSP, Universidade de Sao Paulo, Sao Paulo, SP, BR.; II Centro Neonatal e Terapia Intensiva, Instituto da Crianca e do Adolescente (ICr), Hospital das Clinicas HCFMUSP, Faculdade de Medicina, Universidade de Sao Paulo, Sao Paulo, SP, BR.

The repercussions of infection by the new coronavirus severe acute respiratory syndrome coronavirus 2 (SARS-CoV-2) in pregnant women and newborns are still not well known, with little scientific evidence about their manifestation in the mother-child binomial. Vertical transmission seems possible but has not been documented (1,2). Furthermore, the virus has not been detected in human milk, enabling breastfeeding with protection or the use of expressed milk from the mother (1-3).

Several studies (1,3-7) published to date suggest the possibility of vertical transmission of SARS-CoV-2, but none of them have confirmed this. The virus was not found in amniotic fluid, placenta, umbilical cord blood, and breast milk samples.

Existing data suggest that neonatal transmission of SARS-CoV-2 occurs mainly through droplets from infected caregivers or through contact with contaminated biological material. There are clinical case reports that describe late infection in newborns of mothers infected with SARS-CoV-2. Most of these children presented with mild signs and symptoms, but some developed sepsis and severe respiratory failure (8-10).

We present a case that is similar to most reports in the literature published until now: the patient was a male infant of a 37-year-old mother, with a gestational age of 39 5/7 weeks and no prenatal complications. He was born vaginally, with Apgar scores of 8 and 9 (1st and 5th minute), weight 3.050g, length 51 cm, and head circumference 35 cm. The mother’s amniotic membranes had ruptured 11 hours before delivery.

The patient presented with early respiratory distress, which improved after receiving inhaled oxygen in the first 12 hours of life. Blood was collected to examine for early neonatal sepsis; the results of the examination were normal. Chest radiography showed a left clavicle fracture without pulmonary involvement. The patient was discharged home on the third day of life, on exclusive breastfeeding. His family members complied with the isolation requirements recommended by the Ministry of Health of Brazil (7), and they were asymptomatic. This newborn had had no contact with other patients who had flu-like syndrome.

At 11 days of age, the newborn had two episodes of hyperthermia and mild respiratory distress, according to the mother's report. On examination, the blood count, blood culture results, serum C-reactive protein levels, liquor levels, and type 1 urine examination results were normal. Chest radiography did not reveal any changes in the lung fields. Assessment of nasal and oropharyngeal samples for SARS-CoV-2 by reverse transcription followed by polymerase chain reaction (RT-PCR) revealed positive results.

At age 13 days, the infant was transferred to the Neonatal Center of Instituto da Criança, Hospital das Clínicas, Faculty of Medicine-University of São Paulo, and did not show any respiratory symptoms in room air. He remained in room air throughout hospitalization. He had a hemoglobin saturation greater than 94%, was afebrile, and had a normal heart rate. Pulmonary ultrasonography showed changes compatible with bilateral interstitial involvement (Figure 1). He was mostly breastfed, and was given formula only when breast milk was unavailable.

The spectrum of clinical manifestations of SARS-CoV-2 infection in newborns is still under study. Until now, the presence of the virus in biological materials has not been sufficient to prove its vertical transmission (1,3,6). Case reports have indicated postnatal acquisition of coronavirus disease (COVID-19), as in the case described. The patient's mother did not present respiratory symptoms or show any other clinical evidence of illness. The only finding was rupture of amniotic membranes 11 hours before delivery. The newborn developed early and transient respiratory distress without other complications. Respiratory distress in the neonatal period among neonates with COVID-19-positive mothers may be related to the neonates innate conditions and should not always be attributed to SARS-CoV-2 infection (1,3,8,9).

The patient remained in the exclusive breastfeeding room until discharge home on the third day of life. The binomial was discharged home, where all residents followed social isolation advocated by the state government and did not present any signs or symptoms of COVID-19.

Horizontal transmission seems to be the most likely route of SARS-CoV-2 infection in this case, perhaps through contact with an asymptomatic carrier of the virus.

In the studies (1,3,5,6) conducted until now, the human milk samples tested were negative for the presence of the virus, which made it possible to continue breastfeeding even in symptomatic women. In such cases, it is necessary that the rules of home isolation be respected, suggesting participation of healthy family members in providing care to the newborn during the period of maternal convalescence. The use of a surgical mask, frequent hand hygiene, and observation for warning signs that the newborn may present with are recommended.

The clinical manifestation of COVID-19 among children is characterized by fever, dry cough, tiredness, and a runny nose. Some patients have manifestations in the digestive tract, such as bloating, nausea, vomiting, diarrhea, and abdominal pain. Some cases may present with skin rashes. The progression is generally benign, with complete resolution of the condition within two weeks after symptom onset (11,12).

Descriptions of clinical manifestations in neonates are rarer. A case of severe late-onset neonatal sepsis was recently reported at the University of Texas, with the newborn needing orotracheal intubation and vasoactive drug administration (11).

Much attention is needed at this stage because, to date, the most severe respiratory distress syndromes appear concomitantly with other conditions characteristic of the neonatal period. One should always conduct assessments on a case-by-case basis, classify respiratory failure, and prescribe the best treatment for the patient.

According to Hong et al., (12) the criteria for the diagnosis of COVID-19 among neonates are as follows: thermal instability, hypoactivity, difficulty in feeding, or respiratory distress; alterations of chest radiographs with a single or bilateral ground-glass pattern; diagnosis of COVID-19 in a relative or caregiver of the newborn; and intimate contact with people confirmed or suspected of having COVID-19 or with patients with unexplained pneumonia.

Our patient fulfilled only two the criteria: temperature instability and mild respiratory distress. His hemoglobin saturation at admission was 91% in room air. The tests collected initially did not show any hematological changes, such as leukocytosis or leukopenia, and the platelet count was normal. His serum C-reactive protein level was normal. Because of the presence of fever, urine and cerebrospinal fluid samples were tested, and meningitis and urinary tract infection were ruled out. Ampicillin, cefotaxime, and oseltamivir were administered. The neonate tested positive for COVID-19 24 hours after admission and was transferred to a Neonatal Intensive Care Unit for patients with COVID-19 during the pandemic. The blood samples collected at hospital admission did not show bacterial growth, and the administration of antibiotics was suspended after 48 hours. The newborn was hospitalized for seven days. He remained asymptomatic throughout the hospitalization, with oxygen saturation above 94% and a temperature between 36.5 and 37°C. Disease progression was good, as in other cases (9,10) described in the literature.

COVID-19 has a highly variable clinical presentation, and there are still few case reports on COVID-19 in the neonatal period. Many neonates present with symptoms and signs that overlap with those of diseases typical of this age range. The biggest challenge is to recognize the characteristics of COVID-19 among other manifestations of neonatal diseases and to choose the most appropriate treatment for the patients.

## Figures and Tables

**Figure 1:**
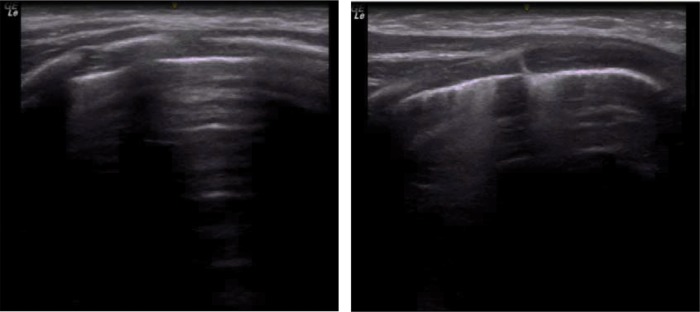
Chest ultrasound of the apex and lateral base of the right hemithorax. The image shows no consolidation and a pattern of interstitial involvement.
